# Higher Serum Free T4 Is Associated With Increased Risk of Mortality and Cerebrovascular Events in Elderly Men

**DOI:** 10.1210/jendso/bvaf121

**Published:** 2025-07-18

**Authors:** Johan Svensson, Claes Ohlsson, Magnus K Karlsson, Åsa Tivesten, Hans Herlitz, Mattias Lorentzon, Catharina Lewerin, Dan Mellström

**Affiliations:** Department of Internal Medicine and Clinical Nutrition, Sahlgrenska Osteoporosis Center, Center for Bone and Arthritis Research, Institute of Medicine, Sahlgrenska Academy, University of Gothenburg, Gothenburg SE-413 45, Sweden; Department of Internal Medicine, Region Västra Götaland, Skaraborg Central Hospital, Skövde SE-541 85, Sweden; Department of Internal Medicine and Clinical Nutrition, Sahlgrenska Osteoporosis Center, Center for Bone and Arthritis Research, Institute of Medicine, Sahlgrenska Academy, University of Gothenburg, Gothenburg SE-413 45, Sweden; Department of Drug Treatment, Region Västra Götaland, Sahlgrenska University Hospital, Gothenburg SE-413 45, Sweden; Department of Clinical Sciences and Orthopedics, Clinical and Molecular Osteoporosis Research Unit, Skane University Hospital, Lund University, Malmö SE-205 02, Sweden; Department of Molecular and Clinical Medicine, Wallenberg Laboratory for Cardiovascular and Metabolic Research, Institute of Medicine, Sahlgrenska Academy, University of Gothenburg, Gothenburg SE-413 45, Sweden; Department of Endocrinology, Sahlgrenska University Hospital, Region Västra Götaland, Gothenburg SE-413 45, Sweden; Department of Molecular and Clinical Medicine/Nephrology, Institute of Medicine, Sahlgrenska Academy, University of Gothenburg, Gothenburg SE-413 45, Sweden; Department of Internal Medicine and Clinical Nutrition, Sahlgrenska Osteoporosis Center, Center for Bone and Arthritis Research, Institute of Medicine, Sahlgrenska Academy, University of Gothenburg, Gothenburg SE-413 45, Sweden; Geriatric Medicine, Department of Internal Medicine and Clinical Nutrition, Institute of Medicine, Sahlgrenska Academy, University of Gothenburg, Gothenburg SE-413 45, Sweden; Department of Hematology and Coagulation, Region Västra Götaland, Sahlgrenska University Hospital, Gothenburg SE-413 45, Sweden; Department of Internal Medicine and Clinical Nutrition, Institute of Medicine, Sahlgrenska Academy, University of Gothenburg, Gothenburg SE-413 45, Sweden; Department of Internal Medicine and Clinical Nutrition, Sahlgrenska Osteoporosis Center, Center for Bone and Arthritis Research, Institute of Medicine, Sahlgrenska Academy, University of Gothenburg, Gothenburg SE-413 45, Sweden; Geriatric Medicine, Department of Internal Medicine and Clinical Nutrition, Institute of Medicine, Sahlgrenska Academy, University of Gothenburg, Gothenburg SE-413 45, Sweden

**Keywords:** serum free T4, serum TSH, mortality, older men, cerebrovascular events, cancer

## Abstract

**Background:**

It is unclear whether thyroid hormone levels are associated with the risk of mortality, cardiovascular disease (CVD) events, or cancer in men with normal TSH.

**Objectives:**

We analyzed if serum free T4 (FT4) or TSH is associated with the risk of mortality, incident CVD events, or cancer in Swedish men.

**Methods:**

Elderly men (n = 1801; mean age 75 years) in the Gothenburg and Malmö subcohorts of the prospective, population-based Osteoporotic Fractures in Men Study-Sweden study were followed for median 12.2 years regarding all-cause mortality [1207 (67%) died] and for median 5.1 years regarding incident CVD events (n = 338) and cancer (n = 249). The statistical analyses included Cox proportional hazards regression with adjustment for covariates including prevalent atrial fibrillation (AF).

**Results:**

Serum FT4 (per SD increase) was associated with increased risk of mortality [men with normal TSH: fully adjusted hazard ratio (HR) 1.23, 95% confidence interval (CI): 1.11-1.35] and incident CVD events (HR 1.25, 95% CI: 1.05-1.48) but not with the risk of cancer. The association between FT4 and CVD events was mainly due to increased risk of cerebrovascular (CBV) events (HR 1.56, 95% CI: 1.24-1.96). Finally, TSH was not associated with the risk of mortality, CVD events, or cancer.

**Conclusion:**

FT4, but not TSH, is a predictor of mortality risk in elderly men. The association between FT4 and elevated risk of CVD events was mostly due to increased risk of CBV events, which remained significant also after adjustment for prevalent AF.

Untreated overt hyperthyroidism results in impaired heart function, increased risk of atrial fibrillation (AF), and increased mortality [1]. Also, overtreatment with levothyroxine has adverse effects of a similar type [2, 3]. However, it has been less clear whether subclinical hyperthyroidism (SHyper), defined as suppressed TSH with free T4 (FT4) and free T3 levels within the normal range, is associated with increased risk of mortality, cardiovascular disease (CVD), and cancer [4].

In study populations comprising both men and women, there have been conflicting results on whether subclinical thyroid dysfunction is associated with the risk of CVD events and mortality [5-10]. A meta-analysis showed that SHyper tended to be associated with elevated relative risks of coronary heart disease (CHD) and CVD mortality [11]. In a Mendelian randomization study, decreased TSH levels in the direction of mild hyperthyroidism were causally associated with increased risk of AF but not with the risk of CVD [12]. In another Mendelian randomization study, there was a causal association between thyroid dysfunction and breast cancer risk [13]. Furthermore, using individual data on 52 674 participants from 10 cohorts, SHyper was associated with increased risk of total mortality, CHD mortality, and AF and marginally also with elevated risk of CHD events [14]. In another analysis (6 cohorts, n = 25 390), SHyper as well as TSH ≥ 10 mIU/L was associated with increased risk of heart failure [15]. This is in line with other results showing that subclinical hypothyroidism (SHypo), defined as TSH ≥ 10 mIU/L, is associated with increased risk of mortality and CVD events [10, 16], whereas the results have been less clear when lower TSH cut-offs have been used [7, 10, 17].

In individual studies, there have been varying results on whether thyroid hormone levels within the normal range are associated with the risk of mortality and CVD events [18-21]. In individuals with TSH within the reference range, studies using individual participant data from multiple cohorts showed that higher FT4 was associated with increased risk of AF [22], whereas TSH levels were not associated with the risk of CHD [23]. Moreover, lower TSH and higher FT4 were marginally associated with increased risk of all stroke and fatal stroke [24]. In a study of 26 cohorts (n = 134 346), higher FT4 within the normal range was associated with increased risk of a composite outcome (CVD events and all-cause mortality) [25].

The consequences of high thyroid function may be different in men and women [25, 26]. The genetic determinants of thyroid hormones [27] and the relation between TSH and FT4 [28] have been different in men and women, which may indicate sex differences in the set points for thyroid hormones. In one study, women had lower optimal healthy ranges of FT4 than men in terms of the risk of mortality and CVD [25]. However, only a few prospective studies have been conducted in study populations consisting solely of men. In a study based on the Osteoporotic Fractures in Men Study (MrOS)-US study (1587 men, 432 deaths), TSH (per SD decrease) or FT4 (per SD increase) was not associated with the risk of all-cause, CVD, or cancer mortality [29]. In contrast, in the Health in Men Study (n = 3885, 837 deaths), men in the highest FT4 quartile had a higher risk of all-cause mortality [30] and major CVD events [31] compared with men in the 3 lower FT4 quartiles. In addition, in the Health in Men study (n = 3836), TSH was associated with increased colon cancer incidence but not with cancer-related deaths [32]. Finally, data based on the Busselton Health Survey (n = 3649) showed that lower TSH and higher FT4 levels were associated with elevated prostate cancer risk [33].

In summary, overt hyperthyroidism and SHyper are associated with increased risk of mortality and dysrhythmias, including AF. However, the associations have been less clear in men with normal TSH. Therefore, in 2 subcohorts of the prospective, population-based MrOS-Sweden study of elderly men, we investigated whether serum FT4 and TSH levels are associated with the risk of mortality, incident CVD events, and incident cancer. We also analyzed whether FT4 or TSH levels were associated with incident CBV or CHD events. We hypothesized that lower TSH and higher FT4 levels would predict the risk of mortality and incident CVD events in the entire cohort and in men with normal TSH.

## Materials and Methods

### Participants and Ethical Considerations

The MrOS is an international, multicenter study. In MrOS-Sweden, men aged 69 to 81 years were randomly identified using national population registers and asked to participate [34]. Of the contacted men, 45% were included. Inclusion criteria were the ability to walk without assistance, provide informed consent, and supply self-reported information. There were no additional inclusion/exclusion criteria. In the present observational study, the Malmö (n = 1005) and Gothenburg (n = 1010) subcohorts of MrOS-Sweden were employed. At baseline (2001-2004), a physical examination was performed, the men answered a questionnaire, and blood samples were drawn. Then, the men were followed until August 31, 2018, in terms of all-cause mortality (henceforth mortality). Regarding incident CVD events and cancer, the follow-up was terminated December 31, 2008.

Serum TSH was analyzed in 92% of the total cohort (n = 1856). However, we excluded 31 men receiving levothyroxine treatment at baseline, which included one man with a previous history of thyroid cancer. In addition, 24 men treated with oral glucocorticoids were excluded. Therefore, the final sample comprised 1801 men. Serum FT4 had been measured in 1722 of these men. In all men (n = 1801) and in the subpopulation of men with normal serum TSH (n = 1637), we explored whether TSH or FT4 levels were associated with the risk of mortality, incident CVD events, and incident cancer. We defined a normal TSH as serum TSH between 0.45 mIU/L and <4.5 mIU/L [35-37] and SHyper as TSH < 0.45 mIU/L in combination with serum FT4 ≤ 22 pmol/L (the upper normal limit of the reference range). SHypo was classified as TSH ≥ 4.5 mIU/L [35-37] together with FT4 in the normal range (12-22 pmol/L). The FT4 and TSH reference ranges were based on laboratory reference ranges.

Informed consent was obtained from the participants. The study was approved by the regional ethics committee in Gothenburg (593-07) and was performed in accordance with the 1964 Helsinki Declaration and its later amendments.

### Covariate Assessment

Body mass index (BMI) was assessed by dividing weight (in kilograms) with height (in meters) squared. Trunk fat mass was assessed using dual-energy X-ray absorptiometry with the Lunar Prodigy DXA (GE Lunar Corp., Madison, WI, USA) in the Malmö cohort and the Hologic QDR 4500/A-Delphi (Hologic, Waltham, MA, USA) in the Gothenburg cohort. Information on current smoking (yes/no), physical activity, and self-reported medical diagnoses [hypertension, diabetes, stroke, myocardial infarction (MI) or angina pectoris] was gathered using a standardized questionnaire. We defined prevalent CVD at baseline as a history of stroke, MI, and/or angina pectoris. Hypertension was classified as either self-reported antihypertensive treatment or systolic blood pressure of ≥140 mmHg in the supine position after 10 minutes’ rest. Prevalent cancer at baseline was defined as a history of any cancer in the Swedish Cancer Registry before baseline (see later discussion). Prevalent AF was established if a participant had a diagnosis before baseline of AF [International Classification of Diseases (ICD)-9 code 427D or ICD-10 code I48] in the Swedish National Patient Register. The Swedish National Patient Register includes the Swedish Hospital Discharge Register, and, from 2001, the register also covers all patients treated in specialized outpatient care in Sweden.

### Assessment of Mortality

In terms of the mortality assessments, the average follow-up time was 10.9 (SD 4.4) years (median 12.2 years, 25th-75th percentiles: 7.6-14.8 years). This follow-up time was defined as the time between the date of the baseline visit (in the period 2001-2004) and death of any cause, date of emigration, or study end (August 31, 2018). Deaths that occurred during the follow-up time were retrieved from the National Cause of Death Register, which is held by the National Board of Health and Welfare in Sweden; all deaths in Sweden are reported to this register.

### Assessment of Incident CVD and Incident Cancer

The follow-up time for the assessments of CVD and cancer was mean 4.8 (SD 1.5) years (median 5.1 years, 25th-75th percentiles: 4.4-5.8 years) and was recorded as the period from the baseline visit to the date of first occurrence of CVD event or cancer, death from any cause, date of emigration, or last data collection (December 31, 2008).

#### Incident CVD events

Causes of death, registered with ICD codes based on the information from death certificates, were available from the Swedish Cause of Death Register from the study start until December 31, 2005. Additionally, causes of death were obtained by evaluations of copies of death certificates for deaths occurring after this date until December 31, 2008. Based on the data from the register/death certificates, information was available regarding deaths caused by CHD (ICD-10 codes I20-I25), stroke (ICD-10 codes I60-I64), or other. In addition, data regarding hospitalization for first acute MI (ICD-10 codes I21-I23), unstable angina (ICD-10 codes I20.0 and I24), revascularization procedure (surgery code FN), stroke (ICD-10 codes I60-I64), or transient ischemic attack (ICD-10 code G45) were obtained from the Swedish Hospital Discharge Register from baseline to December 31, 2008. This combination of data from the Swedish Cause of Death Register and the Swedish Hospital Discharge Register, which includes both fatal and nonfatal events, has previously produced valid estimates of the rates of CHD and stroke [38]. Major CVD events were defined as a composite endpoint of CHD events (hospitalization for acute MI, unstable angina or revascularization, or death from CHD) and cerebrovascular (CBV) events (hospitalization for stroke or transient ischemic attack, or death from stroke).

#### Incident cancer

The cancer diagnoses were retrieved from the Swedish Cancer Registry, held by the National Board of Health and Welfare in Sweden. This register contains information on cancer diagnoses (except for basal cell carcinoma) in the Swedish population since 1958. Prevalent cancer was based on a diagnosis of 140-205 (ICD-7) or C00-C97 (ICD-10) in the Swedish Cancer Registry before baseline. Incident cancer was defined as having a diagnosis of C00-C97 after baseline. Information on cancer stage was not available. In men with prevalent cancer at baseline, data were not available on whether an incident cancer occurred during the follow-up. Therefore, men with a history of previous cancer were excluded from the analyses of the associations between serum FT4 or TSH and the risk of incident cancer.

### Biochemical Methods

All blood samples were collected at baseline (from 2001 to 2004) and stored at −80 °C pending analysis. FT4 and TSH in serum were analyzed in 2008 on the Roche Modular system (Roche Diagnostics Scandinavia AB, Solna, Sweden) [39-41]. The total coefficient of variation was below 10% for both the FT4 assay and the TSH assay. The detection limit was 1.5 pmol/L for the FT4 assay and 0.005 mIU/L for the TSH assay. Extremely sensitive C-reactive protein (CRP) was measured using an ultrasensitive method (Orion Diagnostica, Espoo, Finland). Serum levels of apolipoprotein B (ApoB) and apolipoprotein A1 (ApoA1) were determined by immunoprecipitation enhanced by polyethylene glycol at 340 nm (Thermo Fisher Scientific, Vantaa, Finland). The CRP, ApoB, and ApoA1 analyses were performed on a Konelab 20 autoanalyzer (Thermo Fisher Scientific) with interassay coefficients of variation below 5%.

### Statistical Analyses

SPSS for Windows (version 29.0, IBM Corp., Armonk, NY, USA) was used. Data are given as the mean and SD if not otherwise stated. Between-group differences were assessed using 1-way ANOVA for continuous variables and using chi-square tests for categorical variables.

Hazard ratios (HRs) and 95% confidence intervals (CIs) were assessed using Cox proportional hazards regression analyses. Quadratic terms of serum FT4 or TSH did not contribute significantly to the models (*P* > .05) and were therefore excluded from further analyses, as a linear fit of the data was considered adequate. In the total study population (n = 1801 with serum TSH) as well as in the subpopulation of men having normal serum TSH (n = 1637), we analyzed whether FT4 and TSH as standardized continuous variables (per SD increase in serum FT4 and per SD decrease in serum TSH) were associated with the risk of mortality, incident CVD events, and incident cancer. We adjusted for age and MrOS site in all analyses. Moreover, in model A, to evaluate the independent effect of FT4 or TSH, further adjustments were made for BMI, quartile of physical activity (kilometers walked per day), current smoking (yes/no), hypertension (yes/no), and diabetes mellitus (yes/no). In model B, we additionally adjusted for serum CRP (log-transformed), ApoB/ApoA1 ratio, previous history of CVD (yes/no), and previous history of cancer (yes/no). Thus, as in several previous studies of the association between thyroid function and mortality or CVD events [23-25], we included a covariate reflecting lipid metabolism, and we chose the ApoB/ApoA1 ratio as this ratio has been associated with the risk of major adverse CVD events [42]. In the final model (model C), we also included previous history of AF as a covariate. In addition, we analyzed the risk of mortality, incident CVD events, and incident cancer in the highest FT4 quartile (quartile IV) compared with that in the 3 lower quartiles (quartiles I-III) using Cox proportional hazards regression. We performed additional analyses using Cox proportional hazards regression to determine whether FT4 was associated with the risk of incident CBV or CHD events. Finally, we performed subanalyses in which we added trunk fat mass as a covariate in the previously performed Cox proportional hazards regression analyses. A 2-sided *P* < .05 was considered statistically significant.

## Results

### Baseline Characteristics

Baseline characteristics in the total cohort as well as in men who died or survived during the follow-up are given in Table 1. Men who died during the follow-up were older and had higher trunk fat mass, higher serum CRP, lower serum ApoA1, and lower physical activity (kilometers walked per day) compared with survivors. Current smoking, diabetes, previous history of CVD, previous history of cancer, and previous history of AF were more common in men who died compared with those who survived during the follow-up. Finally, men who died had higher baseline serum FT4 level than men who survived, whereas baseline serum TSH was similar in the 2 groups.

**Table 1.** Baseline characteristics in the total study population, in survivors, and in men who died during the follow-up

Variable	All men (n = 1801)	Survivors (n = 594)	Men who died (n = 1207)	*P^a^*
Age (years)	75.4 (3.2)	74.3 (3.0)	75.9 (3.2)	<.001
BMI (kg/m^2^)	26.4 (3.6)	26.3 (3.2)	26.4 (3.8)	.31
Trunk fat mass (kg)	11.9 (4.9)	11.4 (4.4)	12.1 (5.1)	<.01
CRP (mg/L)	4.6 (10.2)	3.6 (9.6)	5.1 (10.5)	<.01
ApoA1 (g/L)	1.53 (0.30)	1.56 (0.29)	1.52 (0.31)	.01
ApoB (g/L)	1.07 (0.25)	1.09 (0.25)	1.07 (0.25)	.08
ApoB/ApoA1	0.723 (0.205)	0.718 (0.194)	0.726 (0.210)	.44
Physical activity (km walk/day)	3.68 (3.07)	4.09 (3.34)	3.47 (2.91)	<.001
Current smoking, % (n)	9.1 (163)	5.9 (35)	10.6 (128)	.001
Hypertension, % (n)	63.9 (1151)	62.6 (372)	64.5 (779)	.44
Diabetes mellitus, % (n)	10.2 (183)	5.9 (35)	12.3 (148)	<.001
History of cardiovascular disease, % (n)	25.7 (463)	16.0 (95)	30.5 (368)	<.001
History of cancer, % (n)	19.9 (358)	15.3 (91)	22.1 (267)	<.001
History of atrial fibrillation, % (n)	8.1 (146)	4.5 (27)	9.9 (119)	<.001
Serum TSH (mIU/L)	2.61 (3.63)	2.50 (1.94)	2.66 (4.22)	.39
Serum FT4 (pmol/L)	17.1 (3.5)	16.8 (2.2)	17.2 (3.9)	.02

If not otherwise stated, values are given as means (SD). Between-group differences were examined using 1-way ANOVA for continuous variables and chi-square tests for categorical variables.

Abbreviations: ApoA1, apolipoprotein A1; ApoB, apolipoprotein B; BMI, body mass index; CRP, C-reactive protein; FT4, free T4.

^a^ *P*-values between men who survived vs men who died.

Serum FT4 correlated positively with serum ApoA1 (r = 0.06, *P* = .01) and negatively with trunk fat mass (r = −0.07, *P* < .01) and ApoB/ApoA1 ratio (r = −0.05, *P* = .04) but not with age (r = 0.02), BMI (r = −0.04), serum CRP (r = 0.03), serum ApoB (r = 0.00), or physical activity (kilometers walked per day, r = 0.01).

Serum TSH correlated positively with age (r = 0.05, *P* = .03) but not with BMI (r = 0.03), trunk fat mass (r = −0.02), serum CRP (r = 0.01), serum ApoA1 (r = −0.02), serum ApoB (r = 0.01), ApoB/ApoA1 ratio (r = 0.00), or physical activity (kilometers walked per day, r = 0.00).

### SHyper and SHypo

There were only 21 men with SHyper at baseline [serum TSH < 0.45 mIU/L and serum FT4 ≤ 22 pmol/L; 16 (76%) of these men died during the follow-up]. Therefore, we did not perform further analyses in men with SHyper. Moreover, there were 124 men with SHypo at baseline (serum TSH ≥ 4.5mIU/L and serum FT4 = 12-22 pmol/L); 87 (70%) of these men died. Although the number of men with SHypo at baseline was small, we performed Cox proportional hazards regression analyses to evaluate whether the rate of mortality, CVD events, or cancer differed between men having or not having SHypo. However, the risk of mortality was similar in men with or without SHypo [adjustment for age and study site (base model): HR 1.10, 95% CI: 0.89-1.37; fully adjusted (model C): HR 1.06, 95% CI: 0.84-1.34; not shown]. Also, the risks of incident CVD events (model C: HR 0.81, 95% CI: 0.51-1.29) and incident cancer (model C: HR 0.76, 95% CI: 0.42-1.36) were similar in men having or not having SHypo.

### Serum FT4 and Risk of Mortality, Incident CVD, and Incident Cancer

During the follow-up time (from baseline to August 31, 2018; median follow-up 12.2 years), 1207 (67%) of the men died. In all men with measurement of serum FT4 (n = 1722), Cox proportional hazards regression showed that serum FT4 as a standardized continuous variable (per SD increase) was associated with increased risk of mortality in all models (base model: HR 1.16, 95% CI: 1.10-1.22; model C: HR 1.18, 95% CI: 1.08-1.29; Table 2).

**Table 2.** Risk (hazard ratios and 95% confidence intervals) of all-cause mortality, incident CVD, and incident cancer per SD increase in serum FT4 levels

	Total study population*^a^* (n = 1722)	Men with normal serum TSH*^b^* (n = 1564)
All-cause mortality (follow-up until August 31, 2018)		
Deaths, n (%)	1152 (67)	1038 (66)
Base model	1.16 (1.10-1.22)	1.17 (1.12-1.23)
Multivariate model A	1.17 (1.11-1.23)	1.18 (1.12-1.24)
Multivariate model B	1.17 (1.11-1.23)	1.17 (1.11-1.22)
Multivariate model C	1.18 (1.08-1.29)	1.23 (1.11-1.35)
Incident CVD events (follow-up until December 31, 2008)		
Events, n (%)	325 (19)	298 (19)
Base model	1.09 (1.004-1.18)	1.08 (0.998-1.17)
Multivariate model A	1.09 (1.01-1.17)	1.08 (1.0003-1.17)
Multivariate model B	1.10 (1.01-1.19)	1.09 (1.004-1.18)
Multivariate model C	1.21 (1.03-1.41)	1.25 (1.05-1.48)
Incident cancer (follow-up until December 31, 2008)*^c^*		
Cases, n (%)	243 (14)	224 (14)
Base model	1.08 (0.91-1.29)	1.06 (0.87-1.28)
Multivariate model A	1.08 (0.91-1.29)	1.06 (0.88-1.29)
Multivariate model B	1.10 (0.91-1.32)	1.09 (0.90-1.33)
Multivariate model C	1.09 (0.90-1.31)	1.09 (0.89-1.32)

Hazard ratios were calculated using Cox proportional hazards regression, in which serum FT4 was included as a standardized continuous variable (per SD increase).

Base model: adjustment for age and MrOS site.

Multivariate model A: age, MrOS site, body mass index, quartile of physical activity (kilometers walked per day), current smoking (yes/no), hypertension (yes/no), and diabetes mellitus (yes/no).

Multivariate model B: age, MrOS site, body mass index, quartile of physical activity, current smoking, hypertension, diabetes mellitus, serum C-reactive protein (log-transformed), apolipoprotein B/apolipoprotein A1 ratio, previous history of CVD (yes/no), and previous history of cancer (yes/no).

Multivariate model C: age, MrOS site, body mass index, quartile of physical activity, current smoking, hypertension, diabetes mellitus, serum C-reactive protein, apolipoprotein B/apolipoprotein A1 ratio, previous history of CVD, previous history of cancer, and previous history of atrial fibrillation.

Abbreviations: CVD, cardiovascular disease; FT4, free T4; MrOS, Osteoporotic Fractures in Men Study.

^a^Men with measurement of serum FT4.

^b^Men with measurement of FT4 and serum TSH between 0.45 mIU/L and <4.5 mIU/L.

^c^In the analyses of incident cancer, men with a previous history of cancer were excluded.

In terms of the assessments of incident CVD events and incident cancer, the follow-up time was median 5.1 years (from baseline to December 31, 2008). In the entire cohort, serum FT4 (per SD increase) was associated with increased risk of incident CVD events (model C: HR 1.21, 95% CI: 1.03-1.41), whereas there was no association with the risk of incident cancer (Table 2).

In men with normal serum TSH (TSH between 0.45 and <4.5 mIU/L; n = 1564 with serum FT4 measurement), FT4 (per SD increase) was significantly associated with elevated mortality risk in all models (model C: HR 1.23, 95% CI: 1.11-1.35; Table 2). Furthermore, higher FT4 was also associated with increased risk of CVD events in all the adjusted models (model C: HR 1.25, 95% CI: 1.05-1.48), whereas FT4 was not associated with the risk of cancer (Table 2).

### Analyses of Men in the Highest Serum FT4 Quartile

Next, we compared the highest FT4 quartile (quartile IV) with the 3 lower FT4 quartiles (quartiles I-III). Men in the highest FT4 quartile had a higher prevalence at baseline of smoking [quartile IV: n = 46 (12%) vs quartiles I-III: n = 109 (8%); *P* = .03], hypertension [quartile IV: n = 270 (69%) vs quartiles I-III: n = 832 (63%); *P* = .03], previous history of CVD [quartile IV: n = 115 (29%) vs quartiles I-III: n = 321 (24%); *P* = .03], and previous history of AF [quartile IV: n = 50 (12.7%) vs quartiles I-III: n = 96 (7.2%); *P* < .001]. All other baseline characteristics were similar in both groups (data not shown).

**Figure 1.**
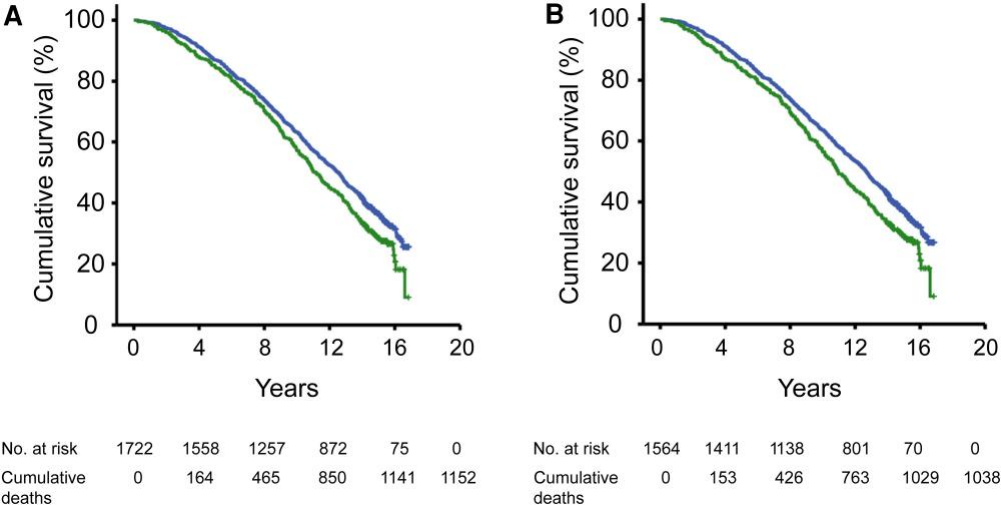
Higher serum FT4 is associated with increased risk of all-cause mortality. Kaplan-Meier survival curves for the risk of mortality in (A) all men with measurement of serum FT4 (n = 1722; quartile IV vs quartiles I-III; log-rank test: *P* < .01) and (B) men with normal serum TSH (between 0.45 and <4.5 mIU/L) and measurement of serum FT4 (n = 1564; quartile IV vs quartiles I-III; log-rank test: P = .001). Green: men in the highest FT4 quartile (quartile IV); blue: men in the 3 lower FT4 quartiles (quartiles I-III).

Abbreviation: FT4, free T4.

In all men with serum FT4 (n = 1722), men in the highest FT4 quartile had elevated risk of mortality (quartile IV vs quartiles I-III: model C: HR 1.20, 95% CI: 1.05-1.39; Table 3). The risk of incident CVD events was also increased in the highest FT4 quartile (vs quartiles I-III: model C: HR 1.30, 95% CI: 1.01-1.67). In contrast, the risk of cancer was unchanged in the highest FT4 quartile (Table 3). Kaplan-Meier survival curves confirmed that the highest FT4 quartile was associated with increased risk of mortality (quartiles IV vs quartiles I-III; log-rank test, *P* < .01; Fig. 1A) and incident CVD events (log-rank test, *P* = .01; not shown).

**Table 3.** Risk (hazard ratios and 95% confidence intervals) of all-cause mortality, CVD, and cancer in the highest serum FT4 quartile compared with the 3 lower FT4 quartiles

	Total study population*^a^* (n = 1722)		Men with normal serum TSH*^b^* (n = 1564)	
	Quartile IV (n = 394) (>75th percentile)	Quartiles I-III (n = 1328) (≤75th percentile)	Quartile IV (n = 360) (>75th percentile)	Quartiles I-III (n = 1204) (≤75th percentile)
All-cause mortality (follow-up until August 31, 2018)				
Deaths, n (%)	287 (73)	865 (65)	262 (73)	776 (64)
Base model	1.24 (1.08-1.41)	1.0 referent	1.27 (1.10-1.46)	1.0 referent
Multivariate model A	1.24 (1.08-1.42)	1.0 referent	1.28 (1.11-1.48)	1.0 referent
Multivariate model B	1.23 (1.07-1.41)	1.0 referent	1.28 (1.10-1.48)	1.0 referent
Multivariate model C	1.20 (1.05-1.39)	1.0 referent	1.25 (1.08-1.45)	1.0 referent
Incident CVD events (follow-up until December 31, 2008)				
Events, n (%)	90 (23)	235 (18)	83 (23)	215 (18)
Base model	1.34 (1.05-1.71)	1.0 referent	1.35 (1.05-1.74)	1.0 referent
Multivariate model A	1.36 (1.06-1.75)	1.0 referent	1.36 (1.05-1.76)	1.0 referent
Multivariate model B	1.30 (1.01-1.67)	1.0 referent	1.30 (1.003-1.70)	1.0 referent
Multivariate model C	1.30 (1.01-1.67)	1.0 referent	1.30 (1.0003-1.70)	1.0 referent
Incident cancer (follow-up until December 31, 2008)*^c^*				
Cases, n (%)	55 (14)	188 (14)	51 (14)	173 (14)
Base model	0.97 (0.71-1.31)	1.0 referent	0.97 (0.71-1.32)	1.0 referent
Multivariate model A	0.93 (0.68-1.27)	1.0 referent	0.94 (0.68-1.31)	1.0 referent
Multivariate model B	0.95 (0.69-1.30)	1.0 referent	0.97 (0.70-1.34)	1.0 referent
Multivariate model C	0.93 (0.67-1.27)	1.0 referent	0.95 (0.69-1.32)	1.0 referent

Hazard ratios were calculated using Cox proportional hazards regression.

Base model: adjustment for age and MrOS site.

Multivariate model A: age, MrOS site, body mass index, quartile of physical activity (kilometers walked per day), current smoking (yes/no), hypertension (yes/no), and diabetes mellitus (yes/no).

Multivariate model B: age, MrOS site, body mass index, quartile of physical activity, current smoking, hypertension, diabetes mellitus, serum C-reactive protein (log-transformed), apolipoprotein B/apolipoprotein A1 ratio, previous history of CVD (yes/no), and previous history of cancer (yes/no).

Multivariate model C: age, MrOS site, body mass index, quartile of physical activity, current smoking, hypertension, diabetes mellitus, serum C-reactive protein, apolipoprotein B/apolipoprotein A1 ratio, previous history of CVD, previous history of cancer, and previous history of atrial fibrillation.

Abbreviations: CVD, cardiovascular disease; FT4, free T4; MrOS, Osteoporotic Fractures in Men Study.

^a^Men with measurement of serum FT4.

^b^Men with measurement of FT4 and serum TSH between 0.45 mIU/L and <4.5 mIU/L.

^c^In the analyses of incident cancer, men with a previous history of cancer were excluded.

In men with normal TSH (n = 1564 with serum FT4), there was also a marked increase in mortality risk in the highest FT4 quartile compared with the 3 lower quartiles (base model: HR 1.27, 95% CI: 1.10-1.46; model C: HR 1.25, 95% CI: 1.08-1.45) (Table 3). Moreover, men in the highest FT4 quartile had an increased risk of CVD events in the base model (HR 1.35, 95% CI: 1.05-1.74), which marginally remained statistically significant in the fully adjusted model (model C: HR 1.30, 95% CI: 1.0003-1.70). Men in the highest FT4 quartile had a similar risk of cancer as those in the lower quartiles (Table 3). Finally, Kaplan-Meier survival curves illustrated that the highest FT4 quartile was associated with increased risk of mortality (quartile IV vs quartiles I-III; log-rank test: *P* = .001; Fig. 1B) and CVD events (log-rank test, *P* = .01; not shown) in men with normal TSH.

### Serum FT4 and Risk of Incident CBV and CHD Events

As there were associations between higher serum FT4 and increased risk of incident CVD events, we performed exploratory analyses using Cox proportional hazards regression to determine whether serum FT4 was associated with the risk of incident CBV and CHD events (Table 4). In the entire cohort, FT4 (per SD increase) was associated with increased risk of CBV events both in the total study population (n = 1722; model C: HR 1.47, 95% CI: 1.19-1.81) and in men with normal TSH (n = 1564; model C: HR 1.56, 95% CI: 1.24-1.96). On the contrary, serum FT4 was not associated with the risk of CHD events (Table 4).

**Table 4.** Risk (hazard ratios and 95% confidence intervals) of incident CBV and CHD events per SD increase in serum FT4 levels

	Total study population*^a^* (n = 1722)	Men with normal serum TSH*^b^* (n = 1564)
Incident CBV events (follow-up until December 31, 2008)		
Events, n (%)	155 (9)	142 (9)
Base model	1.12 (1.04-1.21)	1.12 (1.03-1.21)
Multivariate model A	1.12 (1.04-1.20)	1.11 (1.03-1.20)
Multivariate model B	1.13 (1.04-1.22)	1.12 (1.03-1.21)
Multivariate model C	1.47 (1.19-1.81)	1.56 (1.24-1.96)
Incident CHD events (follow-up until December 31, 2008)		
Events, n (%)	197 (11)	181 (12)
Base model	1.05 (0.92-1.21)	1.06 (0.93-1.21)
Multivariate model A	1.06 (0.92-1.21)	1.06 (0.92-1.21)
Multivariate model B	1.06 (0.91-1.24)	1.07 (0.92-1.23)
Multivariate model C	1.08 (0.88-1.32)	1.11 (0.89-1.40)

Hazard ratios were calculated using Cox proportional hazards regression, in which serum FT4 was included as a standardized continuous variable (per SD increase).

Base model: adjustment for age and MrOS site.

Multivariate model A: age, MrOS site, body mass index, quartile of physical activity (kilometers walked per day), current smoking (yes/no), hypertension (yes/no), and diabetes mellitus (yes/no).

Multivariate model B: age, MrOS site, body mass index, quartile of physical activity, current smoking, hypertension, diabetes mellitus, serum C-reactive protein (log-transformed), apolipoprotein B/apolipoprotein A1 ratio, previous history of cardiovascular disease (yes/no), and previous history of cancer (yes/no).

Multivariate model C: age, MrOS site, body mass index, quartile of physical activity, current smoking, hypertension, diabetes mellitus, serum C-reactive protein, apolipoprotein B/apolipoprotein A1 ratio, previous history of cardiovascular disease, previous history of cancer, and previous history of atrial fibrillation.

Abbreviations: CBV, cerebrovascular disease; CHD, coronary heart disease; FT4, free T4; MrOS, Osteoporotic Fractures in Men Study.

^a^Men with measurement of serum FT4.

^b^Men with measurement of FT4 and serum TSH between 0.45 mIU/L and <4.5 mIU/L.

In further analyses, in the total cohort, men in the highest FT4 quartile had a higher risk of CBV events than men in the 3 lower FT4 quartiles in all models (model C: HR 1.51, 95% CI: 1.06-2.14) (Table 5). Also, in men with normal TSH, the highest FT4 quartile was associated with increased CBV risk (model C: HR 1.50, 95% CI: 1.04-2.16) (Table 5). Finally, Kaplan-Meier survival curves showed that the highest FT4 quartile was associated with increased risk of CBV events (quartile IV vs quartiles I-III; log-rank test, *P* < .01 in the entire cohort and log-rank test, *P* = .01 in men with normal TSH; data not shown).

**Table 5.** Risk (hazard ratios and 95% confidence intervals) of incident CBV events in the highest serum FT4 quartile compared with the 3 lower FT4 quartiles

	Total study population*^a^* (n = 1722)		Men with normal serum TSH*^b^* (n = 1564)	
	Quartile IV (n = 394) (>75th percentile)	Quartiles I-III (n = 1328) (≤75th percentile)	Quartile IV (n = 360) (>75th percentile)	Quartiles I-III (n = 1204) (≤75th percentile)
Incident CBV events (follow-up until December 31, 2008)				
Events, n (%)	48 (12%)	107 (8)	44 (12)	98 (8)
Base model	1.53 (1.08-2.15)	1.0 referent	1.53 (1.07-2.18)	1.0 referent
Multivariate model A	1.54 (1.09-2.18)	1.0 referent	1.53 (1.06-2.20)	1.0 referent
Multivariate model B	1.48 (1.04-2.10)	1.0 referent	1.47 (1.02-2.13)	1.0 referent
Multivariate model C	1.51 (1.06-2.14)	1.0 referent	1.50 (1.04-2.16)	1.0 referent

Hazard ratios were calculated using Cox proportional hazards regression.

Base model: adjustment for age and MrOS site.

Multivariate model A: age, MrOS site, body mass index, quartile of physical activity (kilometers walked per day), current smoking (yes/no), hypertension (yes/no), and diabetes mellitus (yes/no).

Multivariate model B: age, MrOS site, body mass index, quartile of physical activity, current smoking, hypertension, diabetes mellitus, serum C-reactive protein (log-transformed), apolipoprotein B/apolipoprotein A1 ratio, previous history of cardiovascular disease (yes/no), and previous history of cancer (yes/no).

Multivariate model C: age, MrOS site, body mass index, quartile of physical activity, current smoking, hypertension, diabetes mellitus, serum C-reactive protein, apolipoprotein B/apolipoprotein A1 ratio, previous history of cardiovascular disease, previous history of cancer, and previous history of atrial fibrillation.

Abbreviations: CBV, cerebrovascular disease; FT4, free T4; MrOS, Osteoporotic Fractures in Men Study.

^a^Men with measurement of serum FT4.

^b^Men with measurement of FT4 and serum TSH between 0.45 mIU/L and <4.5 mIU/L.

### Serum TSH and the Risk of Mortality, CVD, and Cancer

In all men (n = 1801), Cox proportional hazards regression showed that serum TSH as a standardized continuous variable (per SD decrease) was not associated with the risk of mortality, incident CVD events, or incident cancer (Table 6). Also, in men with normal serum TSH (n = 1637), serum TSH (per SD decrease) was not associated with the risk of mortality, CVD events, or cancer (Table 6).

**Table 6.** Risk (hazard ratios and 95% confidence intervals) of all-cause mortality, incident CVD events, and incident cancer per SD decrease in serum TSH levels

	Total study population (n = 1801)	Men with normal serum TSH (n = 1637)*^a^*
All-cause mortality (follow-up until August 31, 2018)		
Deaths, n (%)	1207 (67)	1090 (67)
Base model	0.99 (0.94-1.05)	1.03 (0.97-1.09)
Multivariate model A	1.00 (0.94-1.05)	1.03 (0.97-1.10)
Multivariate model B	1.00 (0.95-1.06)	1.02 (0.96-1.09)
Multivariate model C	1.00 (0.95-1.06)	1.03 (0.97-1.10)
Incident CVD events (follow-up until December 31, 2008)		
Events, n (%)	338 (19)	311 (19)
Base model	0.97 (0.89-1.06)	1.05 (0.93-1.17)
Multivariate model A	0.97 (0.88-1.06)	1.06 (0.94-1.19)
Multivariate model B	0.99 (0.90-1.09)	1.05 (0.93-1.18)
Multivariate model C	0.99 (0.90-1.08)	1.05 (0.93-1.18)
Incident cancer (follow-up until December 31, 2008)*^b^*		
Cases, n (%)	249 (14)	230 (14)
Base model	1.12 (0.89-1.41)	0.94 (0.83-1.07)
Multivariate model A	1.12 (0.89-1.42)	0.95 (0.83-1.08)
Multivariate model B	1.13 (0.89-1.45)	0.95 (0.83-1.09)
Multivariate model C	1.12 (0.89-1.42)	0.95 (0.83-1.09)

Hazard ratios were calculated using Cox proportional hazards regression, in which serum TSH was included as a standardized continuous variable (per SD decrease).

Base model: adjustment for age and MrOS site.

Multivariate model A: age, MrOS site, body mass index, quartile of physical activity (kilometers walked per day), current smoking (yes/no), hypertension (yes/no), and diabetes mellitus (yes/no).

Multivariate model B: age, MrOS site, body mass index, quartile of physical activity, current smoking, hypertension, diabetes mellitus, serum C-reactive protein (log-transformed), apolipoprotein B/apolipoprotein A1 ratio, previous history of CVD (yes/no), and previous history of cancer (yes/no).

Multivariate model C: age, MrOS site, body mass index, quartile of physical activity, current smoking, hypertension, diabetes mellitus, serum C-reactive protein, apolipoprotein B/apolipoprotein A1 ratio, previous history of CVD, previous history of cancer, and previous history of atrial fibrillation.

Abbreviations: CVD, cardiovascular disease; MrOS, Osteoporotic Fractures in Men Study.

^a^Men with serum TSH between 0.45 mIU/L and <4.5 mIU/L.

^b^In the analyses of incident cancer, men with a previous history of cancer were excluded.

### Subanalyses

We performed subanalyses using Cox proportional hazards regression in which we included trunk fat mass as a covariate. These analyses showed that in the total study population (n = 1722 with serum FT4 measurement), FT4 (per SD increase) was still associated with the risks of mortality (model C and additional adjustment for trunk fat mass: HR 1.15, 95% CI: 1.06-1.26), CVD (model C and in addition trunk fat mass: HR 1.23, 95% CI: 1.05-1.44), and CBV (model C and trunk fat mass: HR 1.50, 95% CI: 1.21-1.85). Furthermore, also in men with normal serum TSH (n = 1564 with serum FT4 measurement), FT4 (per SD increase) was significantly associated with increased risk of mortality (model C and additional adjustment for trunk fat mass: HR 1.20, 95% CI: 1.09-1.33), CVD (model C and in addition trunk fat mass: HR 1.28, 95% CI: 1.07-1.52), and CBV (model C and trunk fat mass: HR 1.61, 95% CI: 1.28-2.02). Finally, both in the entire study population and in men with normal TSH, men in the highest FT4 quartile had higher risks of mortality, CVD, and CBV compared with men in the 3 lower FT4 quartiles after additional adjustment for trunk fat mass (data not shown).

## Discussion

Overt and subclinical hyperthyroidism have been associated with increased risk of mortality and CVD events, but the associations are less clear in elderly men with normal TSH. Here, we used 2 cohorts of the well-controlled MrOS-Sweden, and we excluded men receiving levothyroxine or oral glucocorticoids and one man with previous thyroid cancer. We performed analyses in the total cohort and in men with normal serum TSH, and we found that higher serum FT4 (per SD increase) was associated with increased risk of mortality and incident CVD events, the latter mainly due to increased risk of CBV events. Furthermore, FT4 was not associated with the risk of cancer, and TSH was not associated with the risk of mortality, CVD events, or cancer. Overall, these results demonstrate that serum FT4, but not serum TSH, is a predictor of mortality risk in elderly men. The association between higher FT4 and elevated CVD risk was mainly due to increased risk of CBV, and the latter association remained significant also after adjustment for multiple covariates, including prevalent AF.

In the present study, FT4 (per SD increase) was associated with increased mortality risk in the entire study population as well as in men with normal TSH (TSH between 0.45 mIU/L and <4.5 mIU/L). Furthermore, men in the highest serum FT4 quartile had an approximately 1.25-fold increase in the risk of mortality compared with men in the 3 lower FT4 quartiles. Therefore, our results concur with the results of most [18, 19, 21, 43], but not all [7, 29], previous studies showing that high-normal thyroid function is associated with increased risk of all-cause mortality. This, in turn, gives further support to the notion that high serum FT4 levels, even in the normal range, are detrimental. Of note, low-risk differentiated thyroid cancer is a condition in which suppressive doses of levothyroxine did not affect the risk of tumor recurrence [44]. Therefore, in low-risk thyroid cancer patients, overly high levothyroxine doses should be avoided in order to minimize therapeutic harm [44, 45].

Our study (n = 1801, mean age 75 years) had a longer duration of follow-up (median 12.2 years) and higher mortality rate (1207 deaths) compared with the 3 previous studies that have investigated the associations between thyroid hormones and mortality in study populations consisting only of elderly men. In the MrOS-US cohort (n = 1587, mean follow-up: 8.3 years, 432 deaths), FT4 or TSH levels were not associated with the risk of all-cause mortality, CVD mortality, or cancer mortality [29]. In contrast, in the Health in Men Study (n = 3885, mean follow-up: 6.4 years, 837 deaths), men in the highest FT4 quartile had an elevated risk of all-cause mortality [30]. Furthermore, in another study based on the Health in Men study (3712 men, mean follow-up 9.5 years, 576 CVD deaths), the risk of a composite variable (CVD death, MI, and stroke) was increased in the highest FT4 quartile [31]. In summary, although previous studies performed specifically in men have produced discrepant results, our study with a longer follow-up and higher mortality rate confirms that high-normal FT4 is associated with increased mortality risk in elderly men.

We found that higher FT4 (per SD increase and in analyses of quartiles) was associated with increased risk of incident CVD events. In exploratory subanalyses, we investigated whether this association was due to an elevated risk of incident CBV or CHD events. The analyses demonstrated that higher FT4 was associated with increased risk of CBV, whereas there was no association with the risk of CHD. In most earlier studies, stroke has been included in composite variables reflecting the total number of CVD events and has not been analyzed separately. However, in some accordance with our results, one study found that treatment of thyroid disorders was associated with a decreased risk of ischemic stroke in men but not in women [46]. Additionally, 2 large studies using individual participant data analysis from several cohorts have assessed the association between thyroid hormones in the normal range and the risk of CBV or CHD events [23, 24]. In a study including 17 cohorts (n = 43 598), lower TSH and higher FT4 across the reference range were weakly associated with increased risk of all stroke and fatal stroke [24]. In contrast, in a study of 14 cohorts (n = 55 412), TSH was not associated with the risk of CHD events or CHD mortality [23], and an association with FT4 was only found in one of the subanalyses. In summary, these previous results combined with our results suggest that in men with high-normal thyroid function, the increased risk of CVD events is to a large extent caused by the increased risk of CBV events.

High-normal thyroid function is associated with an increased risk of dysrhythmias and AF [4, 22], the latter being a major risk factor for stroke [22]. At baseline in our study, the prevalence of AF was relatively high in the total study population (8.1%), and prevalent AF was considerably more common in men in the highest FT4 quartile (12.7%) compared with that in the 3 lower FT4 quartiles (7.2%). However, the associations between serum FT4 and the risks of mortality, CVD events, and CBV events remained significant also after adjustment for multiple covariates, including prevalent AF. In fact, several of the associations were more marked after the addition of prevalent AF as a covariate, suggesting that other mechanisms to a large extent underly the observed associations. Thyroid hormones exert multiple functions, including direct and indirect effects on the heart and the vasculature [4, 24, 47], and further studies are needed to clarify which of these effects are of the most importance for the relations between FT4 and the risks of mortality, CVD events, and CBV events.

There are inconsistent results on whether thyroid hormone dysfunction is associated with cancer risk [29, 48-50]. Although several studies have not found any association between TSH or FT4 levels and cancer risk [29, 50], the Health in Men study (n = 3836) showed that TSH was moderately associated with increased colon cancer incidence [32]. Furthermore, in the Busselton Health Survey (n = 3649), lower TSH and higher FT4 were associated with elevated prostate cancer risk [33]. Additionally, one Mendelian randomization study demonstrated a causal association between thyroid dysfunction and breast cancer risk [13]. In a large register-based study from Israel including both sexes (n = 375 635, median follow-up: 10.9 years, 23 808 cancer cases), thyroid hormone levels were associated with the risk of cancer, but the associations were in different directions in individuals above or below 50 years of age [51]. In the present study, we did not find any association between FT4 or TSH levels and cancer risk, but the follow-up time (median 5.1 years) was moderate in terms of incident cancer events. Thus, the associations between thyroid hormone levels and cancer risk, and whether these associations are dependent on age and cancer type, need to be explored in further studies.

In our analyses, serum TSH (per SD decrease) was not associated with the risk of mortality, incident CVD events, or incident cancer. This is in line with the results of an individual participant data meta-analysis (26 cohorts, n = 134 346), in which the association between higher FT4 and increased risk of a composite outcome (CVD events and mortality) was more consistent than the corresponding association for TSH [25]. Although there are some studies in which TSH, but not FT4, has been associated with mortality risk [21], most studies have shown that FT4 is more clearly associated with the risk of CVD events and mortality compared with TSH [18, 19, 30, 52, 53]. Therefore, our results support that in men, FT4 is a more relevant and considerably stronger marker of the effects of thyroid hormones compared with TSH. In some accordance with this finding, the main function of TSH is to regulate the pituitary secretion of T4, whereas T4 and T3 exert the main effects on the peripheral organs. Still, it seems reasonable to assume that measurements of both TSH and FT4 are needed to have a complete assessment of the risk of mortality and incident CVD events.

Strengths of the present study are the large cohort of well-controlled elderly men and the long follow-up time in terms of mortality. We had access to high-quality data based on national registers in Sweden. All blood samples were obtained at baseline, and none of the blood samples were drawn on a clinical indication. We excluded men receiving treatment with levothyroxine or oral glucocorticoids at baseline and men with a previous history of thyroid cancer. However, it was not recorded if the men had any other previous thyroid disorder or if any of the men received treatment at baseline with amiodarone, antithyroid drugs, lithium, or other medications that can affect thyroid function. Moreover, the use of levothyroxine was only available at baseline, and some of the men could therefore have received levothyroxine treatment initiated after baseline. Thyroid hormones and covariates were only measured at baseline, and therefore, we cannot evaluate if there were changes in thyroid hormone levels across the study period. We did not have access to extended follow-up in terms of incident CVD events and cancer, and a study limitation is therefore that the follow-up time (median 5.1 years) regarding these outcomes was moderate. We had access to prevalent AF at baseline, but a study limitation is that data is lacking in terms of incident AF as well as prevalent and incident heart failure. One additional limitation is the use of self-reported questionnaires for some variables, and we cannot exclude the possibility that this could have resulted in an underestimation of the prevalence of smoking. Furthermore, it is not possible to evaluate whether the observed association between serum FT4 and mortality is causal or whether this association represents a nonspecific consequence of illness. Finally, the study population only comprised elderly Caucasian men, which may reduce the generalizability of our findings to other populations.

In summary, this study has a longer follow-up time and higher rate of mortality compared with previous studies consisting only of older men. We show that higher serum FT4 is associated with increased risk of mortality in Swedish men. We also found an association between higher FT4 and elevated risk of CVD events, whereas there was no association with the risk of cancer. Exploratory subanalyses showed that the association between FT4 and CVD events was mainly due to increased risk of CBV events, and the latter association remained significant also after adjustment for prevalent AF. Finally, TSH was not associated with the risk of mortality, CVD events, or cancer. Overall, our findings suggest that if FT4 levels are measured early, preventive actions can be initialized to minimize the risk of mortality and CVD events.

## Data Availability

Restrictions apply to the availability of some or all data generated or analyzed during this study to preserve patient confidentiality or because they were used under license. The corresponding author will on request detail the restrictions and any conditions under which access to some data may be provided.
